# 

**Published:** 1883-04

**Authors:** 


VOL. IV.] TWO DOLLARS A YEAR. SINGLE COPIES 60 CENTS. [No 2.

### THE JOURNAL OF COMPARATIVE MEDICINE AND SURGERY.

### A QUARTERLY JOURNAL OF THE ANATOMY, PATHOLOGY AND THERAPEUTICS OF THE LOWER ANIMALS.

*Edited by* { W. A. CONKLIN, PH.D.

W. H. PORTER, M.D.

Entered New York Post Office as second class matter.

WILLIAM R. JENKINS,
VETERINARY PUBLISHER, BOOKSELLER AND PRINTER,
850 Sixth Ave., Cor. 48th Street,
NEW YORK.

